# Supplementary material for: Toll-like Receptor-Mediated Immunomodulation of Th1-Type Response Stimulated by Recombinant Antigen of Type 2 Porcine Reproductive and Respiratory Syndrome Virus (PRRSV-2)
Source: Viruses. 2023 Mar 17;15(3):775. doi: 10.3390/v15030775 (PMC10057405; doi:10.3390/v15030775)
Supplement: Supplementary file 1 [file viruses-15-00775-s001.zip › viruses-2221973 Supplementary Materials.pdf]

viruses

Supplementary Materials

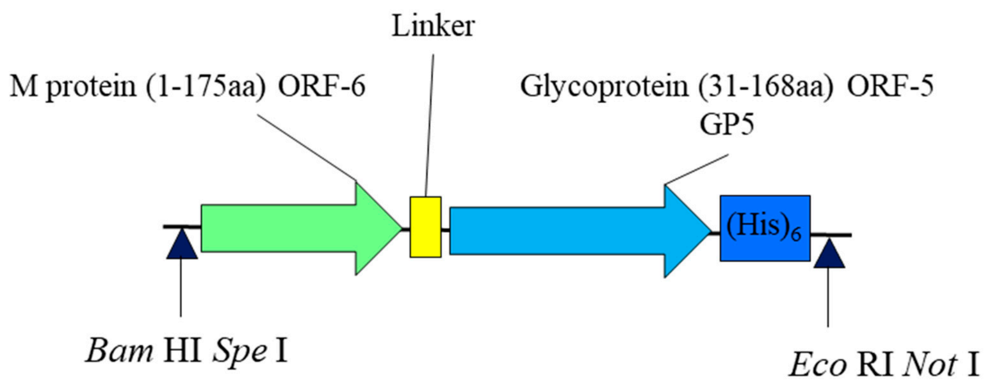

Figure S1. Production of recombinant ORF6L5 (A3) protein. Schematic representation of the ORF6L5 (A3) transfer vector construct. The coding sequence of the PRRSV-2 genes were cloned into baculovirus transfer plasmid between enzyme restriction sites to form the transfer vector. This transfer vector was co-transfected into Hi-5 insect cells along with the baculovirus to form recombinant baculovirus containing PRRSV-2 antigen.

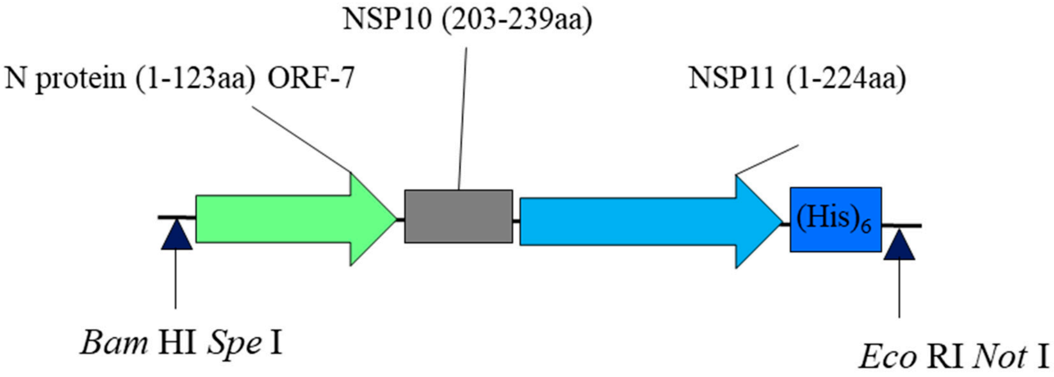

Figure S2. Production of recombinant NLNsp10L11 (A4) protein. Schematic representation of the NLNsp10L11 (A4) transfer vector construct. The coding sequence of the PRRSV-2 genes were cloned into baculovirus transfer plasmid between enzyme restriction sites to form the transfer vector. This transfer vector was co-transfected into Hi-5 insect cells along with the baculovirus to form recombinant baculovirus containing PRRSV-2 antigen.

viruses MDPI

Tabel S1. Primer Sequences for real-time quantitative PCR.

Gene name	GenBank Accesion Number	Primer sequences (5′-3′)	Length (bp)	Annealing Temp (⁰C)
**Porcine *IL-6***	NM_001252429.1	**F:** GCTGCTTCTGGTGATGGCTACTGCC	318	67.78
		**R:** TGAAACTCCACAAGACCGGTGGTGA		66.09
**Porcine *TNF-α***	NM_214022.1	**F:** ATGAGCACTGAGAGCATGATCCG	163	62.66
		**R:** CCTCGAAGTGCAGTAGGCAGA		61.83
**Porcine *Arg-1***	XM_021082747.1	**F:** TGATGTTGACGGACTGGACC	130	59.68
		**R:** ATCTAATCCTGAGAGTAGCCCTGT		60.14
**Porcine *IL-12***	NM_213993.1	**F:** TCAGAAGGCCAAACAAACCCT	134	60.06
		**R:** GGCAACTCTCATTCGTGGCTA		60.41
**Porcine *PPAR-γ***	XM_005669788.3	**F:** TTCTCCAGCATTTCCACTCC	190	57.50
		**R:** AGTTGGAAGGCTCTTCGTGA		58.95
**Porcine *IL-1β***	NM_001302388.2	**F:** TCTGCCCTGTACCCCAACTG	65	61.49
		**R:** CCCAGGAAGACGGGCTTT		59.24
**Porcine *IL-10***	NM_214041.1	**F:** GAACAGCTGCATCCACTTC	150	52.63
		**R:** TAACCCTTAAAGTCCTCCAGC		47.62
**Porcine *CD163***	XM_021091123.1	**F:** ATTCATCATCCTCGGACCCAT	110	58.66
		**R:** CCCAGCACAACGACCACCT		62.48
**Porcine *NF-kB***	NM_001048232.1	**F:** TCGCTGCCAAAGAAGGACAT	101	59.96
		**R:** AGCGTTCAGACCTTCACCGT		61.75

**Porcine *TLR3***	NM_001097444.1	**F:** TGGCCTCTCTCTGAACAATG	139	57.22
		**R:** TCCAAAGAAAGTCGTGTTGC		56.87
**Porcine *TLR4***	NM_001293316.1	**F:** CAGATAAGCGAGGCCGTCATT	113	60.54
		**R:** TTGCAGCCCACAAAAAGCA		59.10
**Porcine *TLR7***	NM_001097434.1	**F:** GGGAAAGCTCCAGTATCTGC	101	58.04
		**R:** TGACGCTTCTGGAACAGTTG		58.42
**Porcine *TLR8***	XM_013985896.2	**F:** AAAAAGCACGTCCCTGAAAGAA	78	58.98
		**R:** TACCTGTCATCTTGGGCATT		56.50
**Porcine *TLR9***	XM_005669564.3	**F:** GCCATTACTAGGGAGGTGGA	111	58.20
		**R:** CTTGCAGTTTGGCATGAAGT		57.19
**Porcine *PI3K***	XM_021086552.1	**F:** GTGACTGACTGTGTAAGCCCA	168	59.93
		**R:** GACTGGCACCTAGAACGTGA		59.40
**Porcine *PKCδ***	XM_021068966.1	**F:** GCAGGGATTAAAGTGTGAAG	151	54.29
		**R:** AACCTCCGACTCTGACTTTC		56.89
**Porcine *PKD-1***	XM_021085680.1	**F:** ACAGTCCCGCCGTCCAG	77	61.42
		**R:** CACAGCCGAGAAGCCGATC		60.88
**Porcine *BCL-3***	NM_001315716.1	**F:** CGACGCGGTGGACATTAAG	72	59.00
		**R:** ACCATGCTAAGGCTGTTGTTTTC		59.00
**Porcine *p65/RELA***	NM_001114281.1	**F:** GGAACACGATGGCCACTTG	96	59.12
		**R:** AAGAGGACATCGAGGTGTATTTCAC		60.62
**Porcine *NFATC1***	NM_214161.1	**F:** ATCTCAGCTGTTGGGTCAGC	809	60.04
		**R:** AGTGAGGGTGAGTGGTCCAG		60.00
**Porcine *β-actin***	XM_021086047.1	**F:** GGATGCAGAAGGAGATCACG	130	58.14
		R: ATCTGCTGGAAGGTGGACAG		59.38
